# Heterogeneous programmed death-ligand 1 expression in gastric cancer: comparison of tissue microarrays and whole sections

**DOI:** 10.1186/s12935-020-01273-0

**Published:** 2020-05-24

**Authors:** Min Ye, Dan Huang, Qiongyan Zhang, Weiwei Weng, Cong Tan, Guangqi Qin, Wenhua Jiang, Weiqi Sheng, Lei Wang

**Affiliations:** 1 Department of Pathology, Fudan University Shanghai Cancer Center, 270 Dong’an Road, Shanghai, 200032 China; 2 Department of Oncology, Shanghai Medical College, Fudan University, Shanghai, 200032 China; 3 Department of Pathology, Zhongshan Hospital, Fudan University, Shanghai, 200032 China

**Keywords:** PD-L1, Gastric cancer, Surgical specimen, Biopsy specimen, Tissue microarray, Heterogeneity

## Abstract

**Background:**

Programmed death-ligand 1 (PD-L1) expression determines the eligibility for anti-PD-1 treatment in patients with advanced gastric cancer, but evidence indicates that PD-L1 staining is heterogeneous. Patients who are ineligible for radical surgery could be tested for PD-L1 expression with biopsy staining, but it is unclear if a small biopsy is representative of the PD-L1 status of the whole tumor. The aim of our study was to determine how many biopsy specimens are needed to accurately reflect the objective status of PD-L1 expression in whole sections.

**Methods:**

We built tissue microarrays (TMAs) as substitutes for core biopsies, collecting 6 cores per case from 152 gastric cancer specimens. All of the slides and TMAs underwent PD-L1 immunohistochemical staining, and PD-L1 expression in at least 1% of tumor cells or immune cells was defined as positive.

**Results:**

It was necessary to randomly select multiple cores from TMAs to reach a suitable agreement rate (> 90%) and Cohen’s κ value (> 0.8) between TMAs and whole sections. We defined the PD-L1 staining status from the whole section as the standard. The evaluation of five randomly selected cores from TMAs agreed well with the evaluation of whole sections. The sensitivity, specificity and the area under the curve (AUC) of the receiver-operating characteristic (ROC) were 0.93, 0.92, and 0.922 (95% confidence interval (CI) 0.863–0.982), respectively.

**Conclusions:**

We conclude that PD-L1 expression among TMA samples had different degrees of relevance to the corresponding surgical specimens, which indicates that at least five biopsies might be necessary to characterize patients taking anti-PD-1 treatment.

## Highlights

- PD-L1 expression is heterogeneous in gastric cancer.
- Heterogeneity of PD-L1 expression results in inconsistency in the PD-L1 staining status between biopsies and surgical specimens.
- We suggest that five core biopsy specimens and a cutoff of 1% are necessary; this method could reach high concordance with whole sections.
- We recommend that for inoperable gastric cancer patients, more than five biopsy specimens are needed to assess the status of PD-L1 expression.

## Background

Programmed death ligand 1 (PD-L1) aroused the interest of researchers when immunotherapy became a popular antitumor treatment. PD-L1, expressed mainly in activated T-cells and sometimes in B lymphocytes and natural killer cells, is the primary ligand of Programmed death 1 (PD-1) [1]. Numerous studies have shown that PD-L1 is expressed in cancer cells [2]. Once PD-1 on immune cells interacts with PD-L1 on the surface of tumor cells, the carcinoma can be misidentified as a part of the body [3]. The PD-1/PD-L1 axis is an important immune checkpoint and can aid in tumor escape from immune monitoring.

Pembrolizumab, a kind of immune checkpoint inhibitor that can block the interaction of PD-1 and PD-L1, was approved by the Food and Drug Administration (FDA) in 2014 [4]. Since then, a large number of clinical trials on patients with non-small-cell lung carcinoma (NSCLC), renal cell carcinoma (RCC), head and neck squamous cell carcinoma, gastric cancer and so on have been designed to verify the safety and efficacy of pembrolizumab [5–7]. In the KEYNOTE-012 and KEYNOTE-059 studies, the results showed that patients taking pembrolizumab have longer overall survival and progression-free survival than control patients [8, 9]. These two trials also showed that the expression of PD-L1 in tumors was a strong predictor of the benefits of anti-PD-1 therapy. Especially in KEYNOTE-059, the objective response rate (ORR) of PD-L1-positive tumors was 15.5%, which was much higher than that of PD-L1-negative tumors [9]. Studies on various tumors, such as NSCLC, melanoma, and renal cell cancer, also found that patients with PD-L1-positive tumors obtained better treatment effects than PD-L1-negative patients [10, 11].

Stomach carcinoma is one of the leading causes of cancer death. Many patients are diagnosed when tumors are inoperable [12]. Encouragingly, the FDA granted accelerated approval to pembrolizumab for advanced gastric cancer on September 22, 2017 [10]. For patients with advanced tumors, the expression of PD-L1 can be detected only by endoscopic biopsy tissue. However, PD-L1 expression is heterogeneous in tumor cells and immune cells (including lymphocytes, macrophages and dendritic cells) [13]. Munari et al. [14] and Erik et al. [15] observed this phenomenon in lung cancer and breast cancer, respectively. Ilie et al. [16] compared PD-L1 expression between the specimens of preoperative biopsy and the corresponding resections in 160 NSCLC patients and found significant discordance between the biopsy and resection samples (the overall discordance rate = 48% and κ value = 0.218). In gastric cancer, can a small tissue sample acquired by a gastroscope represent the actual status of the whole tumor?

To our knowledge, a study comparing PD-L1 expression between core biopsies and surgical resection specimens in gastric cancer is still lacking. In this study, we used tissue microarrays (TMAs) as substitutes for core biopsies to identify how many core biopsies were needed to reflect the actual PD-L1 expression status of resection specimens. By comparing the consistency of TMAs and whole tumor sections, we tried to define the number of biopsy specimens that are needed to closely reflect the actual PD-L1 expression status of the resection specimens.

## Materials and methods

### Tumor specimens

One hundred and fifty-two cases of gastric carcinoma were collected from the records from the Department of Pathology, Fudan University Shanghai Cancer Center. All of these patients underwent radical surgery for gastric cancer at the Fudan University Shanghai Cancer Center from 2010 to 2011, and none of the patients had undergone previous chemotherapy treatment. These 152 cases were selected from our previous study [17]. Among these cases, 114 cases were positive for PD-L1 expression in the whole section, while 38 cases were negative. Ethical consent for this study was obtained from the Clinical Research Ethics Committee of Fudan University Shanghai Cancer Center.

### TMA construction

All the cases were reviewed by two pathologists, and the histological diagnoses were confirmed without discrepancies. A representative paraffin block of every case was selected for the construction of TMAs. For each block, six cores with a diameter of 2 mm were randomly obtained from the tumor. A total of 912 cores of gastric cancer were collected to construct eight TMA blocks.

### Immunohistochemistry (IHC) staining and evaluation

Four-μm sections of TMA were cut and deparaffinized according to standard histological techniques. An immunohistochemical study was performed with the automated immunohistochemical stainer (Ventana, Bench Mark ULTRA). The primary antibody, anti-PD-L1 antibody (Cat. No. ab205961, clone 28-8, Abcam, Cambridge, MA) diluted at 1:50 and incubated at 36 °C for 32 min after antigen retrieved with ULTRA cell conditioning solution (ULTRA CC1, Cat. No. 950-224, Ventana). Then the sections were stained with the OptiView DAB IHC Detection Kit (Cat. No. 05269806001, Ventana) according to the manufacture’s instruments. After washed with PBS, the sections were counter-stained with hematoxylin, dehydrated and mounted. For negative controls, the primary antibody was omitted and was replaced by nonspecific immunoglobins. The appropriate specificity and sensitivity of the antibody for PD-L1 staining was determined using human placenta as a positive control. PD-L1 expression showed membranous staining and/or cytoplasmic staining. The proportion of immunostained cells was evaluated among tumor cells and tumor-infiltrating immune cells (TIICs). Patients with at least 1% PD-L1 staining among tumor cells or immune cells were considered positive.

### Statistical methods

Statistical analysis was performed using the SPSS 23.0 software package (SPSS Inc., Chicago, USA). The Chi-squared test was used to evaluate contingency tables, and receiver-operating characteristic (ROC) curves were used to evaluate the ability of TMA samples to reflect the actual PD-L1 expression status of the resection specimens. Cohen’s κ was calculated to evaluate the consistency between observers. In all tests, a *P* value < 0.05 was considered significant.

## Results

### Patient characteristics

The mean and median age of the patients was 60.6 and 60.0 years old (range from 32 to 84 years old). 104 patients (68.4%) were male and 48 patients (31.6%) were female. Tumor sizes ranged from 2.2 to 15.0 cm, the mean and the median size were 5.1 cm and 4.0 cm respectively. In this cohort, 14 cases (9.2%) were in pathological tumor-node-metastasis (pTNM) stage I, 54 cases (35.5%) were in pTNM stage II, 79 cases (52.0%) were in pTNM stage III and 5 cases (3.3%) were in pTNM stage IV. The clinicopathological characteristics of this cohort are summarized in Table 1.

**Table 1:** Clinicopathological characteristics of gastric cancer patients

Clinicopathological characteristic	N = (%)
Age (years)	
< 60	69 (45.4%)
≥ 60	83 (54.6%)
Gender	
Male	104 (68.4%)
Female	48 (31.6%)
Size (cm)	
< 5	86 (56.6%)
≥ 5	66 (43.4%)
Differentiation	
Well and moderately	18 (11.8%)
Poorly and others	134 (88.2%)
Lauren’s histological classification	
Intestinal	82 (53.9%)
Diffused	37 (24.3%)
Mixed	25 (16.4%)
Indeterminate	8 (5.3%)
Depth of invasion	
T1	7 (4.6%)
T2	22 (14.5%)
T3	45 (29.6%)
T4	78 (51.3%)
Lymph node metastasis	
N0	48 (31.6%)
N1	33 (21.7%)
N2	30 (19.7%)
N3	41 (27.0%)
Vascular invasion	
Negative	68 (44.7%)
Positive	84 (55.3%)
Nerve invasion	
Negative	75 (49.3%)
Positive	77 (50.7%)
pTNM stage	
I	14 (9.2%)
II	54 (35.5%)
III	79 (52.0%)
IV	5 (3.3%)

### Heterogeneous PD-L1 staining and interobserver variability

PD-L1 was detected in the tumor cells and/or TIICs with variable intensities and proportions. Whole sections from total of 114 cases were evaluated as positive, while the whole sections from 38 cases were negative. Among the positive cases, 12 of the 114 cases (10.5%) showed PD-L1 expression in TCs, and 57 cases (50.0%) had positive PD-L1 staining in TIICs. In addition, 45 cases (39.5%) showed PD-L1 expression in both TCs and TIICs. In the assessment of PD-L1 expression in whole sections, discrepancy was observed between the two pathologists (Dan Huang and Qiongyan Zhang) in four cases (Cohen’s κ = 0.930, 95% confidence interval (CI) 0.861–0.999). After consensus, two cases were scored as positive, and the other two were scored as negative.

Several cores were lost during TMA construction and immunohistochemical staining. A total of 124 cases remained with six cores, 7 cases remained with five cores, 9 cases remained with four cores, 6 cases remained with three cores, 2 cases remained with two cores, and 1 case remained with one core. All six cores were lost from 3 cases. Regarding the core-based PD-L1 expression assessment, the two pathologists (Dan Huang and Qiongyan Zhang) obtained different results for 29 cores in 11 cases (Cohen’s κ = 0.915, 95% CI 0.886–0.944). A third pathologist (Lei Wang) helped to achieve consensus. Finally, 7 cores were classified as negative, and the remaining 22 cores were positive (Additional file 1: Table S1).

**Fig. 1:**
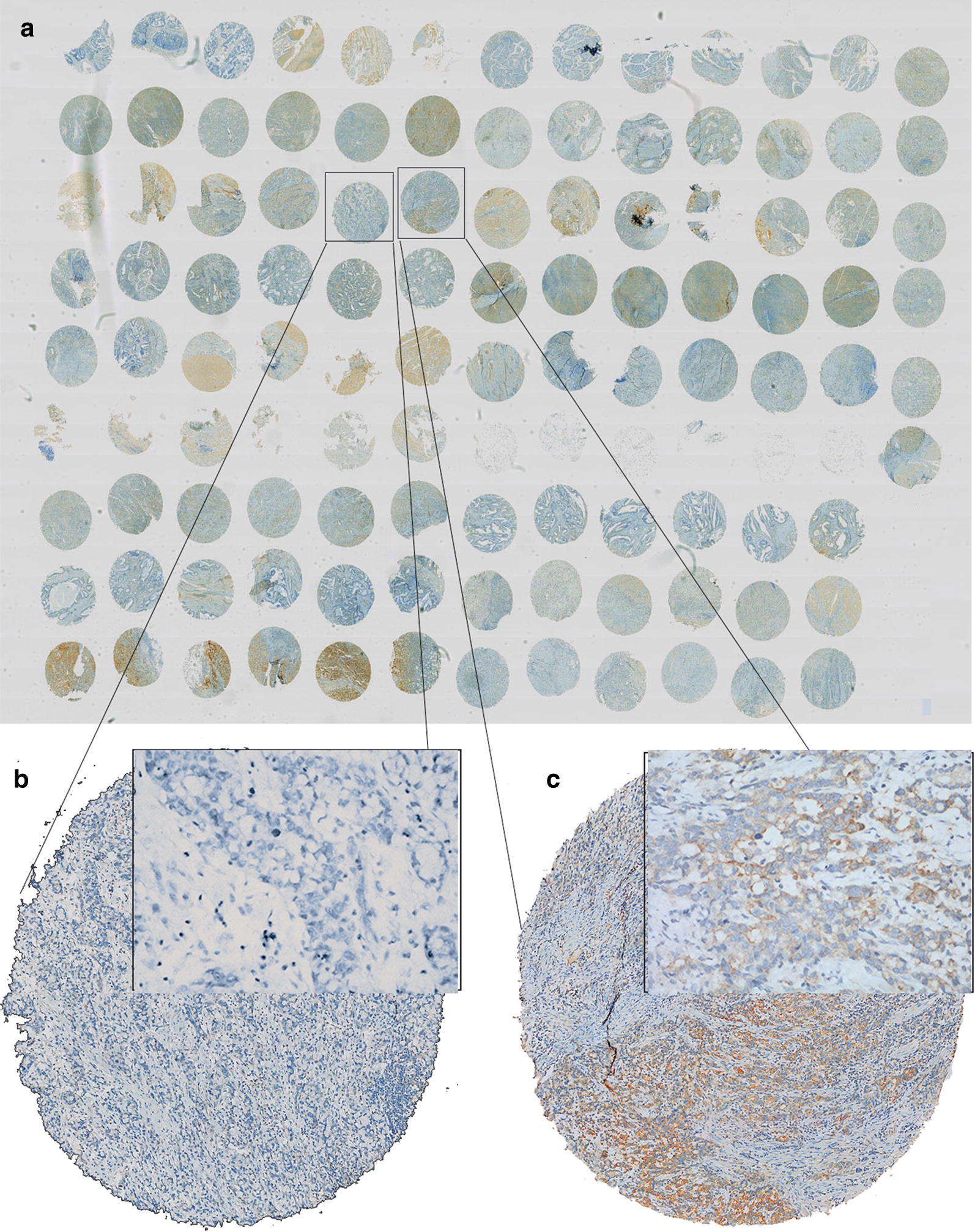
PD-L1 immunohistochemical expression. An overview of an immunohistochemical staining TMA. **b**, **c** Are two cores of the TMA from the same patient. **b** Negative staining, and **c** shows positive staining. Magnification **a**: ×2.5; **b**: ×4 (×200 for insert); **c**: ×4 (×200 for insert)

**Fig. 2:**
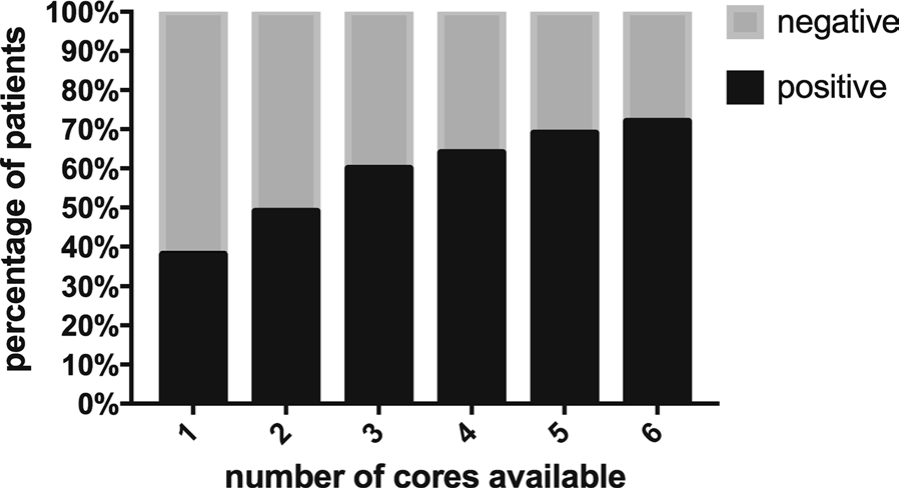
PD-L1 staining status according to the number of cores available

PD-L1 staining heterogeneity was observed in gastric cancer (Fig. 1), which resulted in variations in the PD-L1 staining status in different cores from the same tumor. Thus, PD-L1 positivity rates varied according to the number of cores. When one core from each case was considered, the percentage of PD-L1-positive cases was 38% (56/149). When a second core was available on the TMAs, the positive percentage was 49% (73/148), which represented an increase of more than 10%. Subsequently, the positive percentage increased to 60% (87/146) when three cores were available on the TMAs. The positive rates were 64% (89/140), 69% (90/131) and 72% (89/124) when four, five and six cores were considered, respectively (Fig. 2). The positive rate when six cores were considered on the TMAs was closest to the positive percentage of the whole sections, which was 75% (114/152).

The final results demonstrated that there were inconsistencies between microarrays and whole sections in six cases. Among the 114 PD-L1-positive cases, two cases showed negative PD-L1 expression on the cores of TMAs. The other four inconsistencies occurred in cases that were negative on whole sections. Among these four cases, one case had two positive cores, and the other three had only one positive core. Therefore, the four cases were evaluated as positive on TMAs.

### Determination of the minimal number of cores to represent whole sections

A total of 90 cases had the same results for TMAs with one core and whole sections, with a concordance rate of 60.4% (95% CI 52.5–68.3%) and a κ value of 0.294 (95% CI 0.188–0.400). When two cores in the TMAs were considered, the concordance rate was 72.3% (95% CI 65.0–79.6%), and the κ value was 0.450 (95% CI 0.327–0.573). When the corresponding resection samples were compared to the TMAs with three available cores, the concordance rate was 82.2% (95% CI 75.9–85.5%), and the κ value was 0.607 (moderate agreement). When four cores were considered, the concordance rate was 85.7% (95% CI 79.8–91.6%), with a κ value of 0.672 (95% CI 0.543–0.801). The diagnostic concordance rates were 92.4% (95% CI 87.8–97.0%) and 95.2% (95% CI 91.3–99.0%) when five and six cores were considered on TMAs, respectively, and the κ values were 0.818 (95% CI 0.710–0.926) and 0.883 (95% CI 0.790–0.975), respectively (Table 2). Our results showed that TMAs with five cores were highly consistent with the whole sections, as the concordance rate was more than 90%, and the κ value was above 0.8 (almost perfect agreement).

**Table 2:** Concordance between whole sections and paired TMAs of PD-L1 expression

TMAs	Whole sections			Agreement rate (95% CI)	Cohen’s κ value
No. of cores	Positive	Negative	Total		
1					
Positive	54	2	56	60.4% (52.5–68.3%)	0.294 (0.188–0.400)
Negative	57	36	93		
Total	111	38	149		
2					
Positive	71	2	73	72.3% (65.0–79.6%)	0.450 (0.327–0.573)
Negative	39	36	75		
Total	110	38	148		
3					
Positive	85	2	87	82.2% (75.9–85.5%)	0.607 (0.478–0.736)
Negative	24	35	59		
Total	109	37	146		
4					
Positive	86	3	89	85.7% (79.8–91.6%)	0.672 (0.543–0.801)
Negative	17	34	51		
Total	103	37	140		
5					
Positive	87	3	90	92.4% (87.8–97.0%)	0.818 (0.710–0.926)
Negative	7	34	41		
Total	94	37	131		
6					
Positive	85	4	89	95.2% (91.3–99.0%)	0.883 (0.790–0.975)
Negative	2	33	35		
Total	87	37	124		

*CI* confidence interval

Table 3 summarizes the sensitivity, specificity, and ROCs by taking the PD-L1 staining status of the whole section as the standard. We found that increasing numbers of core biopsies led to a marked increase in the sensitivity (from 0.49 with one core to 0.98 with six cores); the sensitivity reached 0.93 (87/94) when five cores were considered in TMAs. In contrast, we noted that increasing numbers of core biopsies corresponded to a decrease in specificity (from 0.95 with fewer than four cores to 0.89 with six cores), reflecting the growing number of false-positive cases. Since many fewer cells were evaluated on cores than on whole slides, it was possible that $\ge 1\%$ of cells were positive on cores but that less than 1% were positive on the whole section. If we defined PD-L1 positivity based on one core that was positive, the tumor would be diagnosed as PD-L1-positive. And if we defined PD-L1 positivity based on at least two positive cores, the number of false-positive cases would decrease, but the number of false-negative cases would increase. The area under the curve (AUC) of the ROC increased when more cores were available (from 0.717 with one core to 0.934 with six cores). We observed a better value of the AUC (> 0.9) when more than four cores were considered. The AUC was 0.922 (95% CI 0.863–0.982) when we compared the PD-L1 staining status on the TMAs that included five cores with that of whole sections.

**Table 3:** Sensitivity, specificity and ROC AUC according to different number of cores

Cores	Cases	Sensitivity	Specificity	Roc AUC (95% CI)
1	149	0.49	0.95	0.717 (0.633–0.801)
2	148	0.65	0.95	0.796 (0.723–0.870)
3	146	0.78	0.95	0.863 (0.799–0.927)
4	140	0.83	0.92	0.877 (0.811–0.943)
5	131	0.93	0.92	0.922 (0.863–0.982)
6	124	0.98	0.89	0.934 (0.873–0.996)

*ROC* receiver operating characteristic, *AUC* area under the curve

With a cutoff of 1%, evaluating PD-L1 expression in five cores on TMAs could attain high concordance with the results from whole slides. With the evaluation of five cores, the sensitivity, specificity and AUC were 0.93, 0.92 and 0.922 (95% CI 0.863–0.982), respectively.

## Discussion

PD-L1 staining is heterogeneous within most tumors [18]. The heterogeneous distribution of PD-L1 staining intensity can be observed in tumor tissue sections. PD-L1 expression is strong and intense in some areas, while other areas lack PD-L1 expression. The heterogeneity of PD-L1 expression can also be observed in the different histological components of the same tumor. Gagné et al. [19] found that pulmonary adenocarcinoma with solid and micropapillary patterns showed higher PD-L1 expression than pulmonary adenocarcinoma with lepidic, acinar, and papillary patterns. PD-L1 expression in melanoma and colorectal carcinoma was frequently discordant between primary and metastatic tumors [20, 21]. Furthermore, the expression of PD-L1 can change during chemotherapy in gastric cancer patients [22]. Kang et al. [23] showed that the expression of PD-L1 is not related to the efficacy of immunotherapy, as responses could also be observed in PD-L1-negative patients. It is likely that the PD-L1 expression of some tumors could be misclassified due to expression heterogeneity and other factors. An increasing number of studies have found diverse factors that predict favorable outcomes for PD-1/PD-L1 blockade, such as the tumor mutational burden and mismatch-repair status [24, 25]. Nevertheless, PD-L1 expression on tumors was still considered a biomarker for anti-PD-1 treatment [26]. In the KEYNOTE012 and KEYNOTE028 trials, researchers enrolled only PD-L1-positive patients for anti-PD-1 therapy [8, 27]. In addition, several studies have reported that the responses were higher in patients with PD-L1-positive tumors [9, 28].

To date, the cutoff for PD-L1 positivity has not been defined. Different cutoffs have been used in different studies. Topalian et al. [11] chose 5% as the PD-L1 positivity cutoff to assess the role of PD-L1 expression in the response to an anti-PD1 antibody therapy in NSCLC, melanoma, castration-resistant prostate cancer and renal cell cancer. Chen et al. [29] used 10% as the PD-L1 positivity cutoff in pancreatic cancer. For clinicians, the major concern is to avoid omitting patients who might benefit from an effective immunotherapy. Therefore, the definition of PD-L1 positivity as at least 1% of tumor cells or immune cells expressing PD-L1 could be reasonable. The Phase I/II CheckMate-032 trial used a 1% PD-L1 positivity cutoff and revealed that the ORR was higher in PD-L1-positive patients than in negative patients [30]. Moreover, the investigators of KEYNOTE 012 also chose 1% as a cutoff, and a high objective response was also observed [8]. Another clinical trial, KEYNOTE-028, defined PD-L1 positivity as $\ge 1{\text{\% }}$ PD-L1-positive cells (including tumor cells, lymphocytes and macrophages), enrolled advanced solid tumors, and confirmed that the expression of PD-L1 was associated with clinical efficacy and predicted the response to pembrolizumab [27]. Compared with traditional chemotherapy, anti-PD-1/PD-L1 therapy results in clinically meaningful improvements in survival [23]. Therefore, we set 1% as the cutoff for PD-L1 staining in tumor cells or immune cells to discriminate between positivity and negativity.

The heterogeneity of PD-L1 expression may lead to discrepancies in PD-L1 expression in surgical samples and their matched biopsy specimens. In many advanced gastric cancer patients with unresectable tumors, the only specimens available for biomarker assessment are biopsy specimens. However, because of the heterogeneity of PD-L1 expression, PD-L1 expression in biopsy specimens might not be representative of the status of the entire tumor. Some studies showed that it could be necessary to obtain multiple biopsy samples to enhance the predictive value of PD-L1 expression [16, 31–34]. The minimum number of biopsy specimens for concordance with whole tumor slides is a matter of great concern to clinicians. The study carried out by Matsumoto et al. [34] indicated that the concordance rates for the positive frequency of PD-L1 expression between resected and fine needle aspiration (FNA) specimens were lower than 60%. A study on head and neck squamous cell carcinoma also found that intratumor heterogeneity decreases the utility of PD-L1 expression from a single tumor biopsy as a predictive biomarker [33]. Several studies on lung cancer have explored the reliability of small biopsy samples compared with resected specimens for the determination of PD-L1 expression and have examined the specific number of biopsies that are needed [14, 16, 35]. We constructed TMAs as substitutions for diagnostic biopsies, collecting six cores for each case from a total of 152 cases, and we compared the staining results with those obtained for the whole tumor sections. To the best of our knowledge, this is the first study comparing the PD-L1 expression of TMAs and whole sections in gastric adenocarcinomas. The sensitivity and concordance rates of a single TMA core to predict the PD-L1 status of the whole tumor were very low (0.49 and 60.4%, respectively). Our data highlight the fact that a negative result on a small biopsy could be the consequence of a sampling effect. With fewer than five core biopsy specimens, the sensitivity, AUC and consistency rate were not satisfactory (< 0.9), and these values increased with an increase in the number of cores that were evaluated from the TMAs. The chance of obtaining a false-negative result for PD-L1 expression decreased with an increasing number of biopsies. Our results indicated that five core biopsy specimens were necessary to reach an AUC, sensitivity and concordance rate higher than 0.9 and a Cohen’s κ value above 0.8. We used multipoint gastric cancer specimens as a model that could provide more information to assess intratumoral heterogeneity, and TMAs were reasonable substitutions for biopsies [14]; thus, we suggest that five tumor biopsies are necessary to reflect the actual PD-L1 expression status of whole gastric tumors.

## Conclusions

PD-L1 expression is heterogeneous in gastric cancer. When evaluating PD-L1 expression using biopsy specimens, we suggest that five core biopsy specimens are necessary with a positivity cutoff of 1% to reach high concordance with the results from whole sections. Therefore, we believe that for inoperable gastric cancer patients, more than five biopsy specimens are needed to allow clinicians to identify patients who would benefit from immunotherapy.

## Supplementary information

**Additional file 1: Table S1.** List of inconsistent cases between pathologists Huang Dan and Qiongyan Zhang for which a third evaluation by pathologist Lei Wang was essential.

## Data Availability

The datasets used and/or analyzed during the current study are available from the corresponding author on reasonable request.

## Abbreviations

PD-L1: Programmed death ligand 1
PD-1: Programmed death 1
FDA: Food and Drug Administration
TCs: Tumor cells
TIICs: Tumor-infiltrating immune cells
NSCLC: Non-small-cell lung carcinoma
RCC: Renal-cell carcinoma
ORR: Objective response rate
TMAs: Tissue microarrays
IHC: Immunohistochemistry
ROC: Receiver operating characteristic
AUC: Area under the curve
FNA: Fine needle aspiration
