# Supplementary material for: Heterogeneous programmed death-ligand 1 expression in gastric cancer: comparison of tissue microarrays and whole sections
Source: Cancer Cell Int. 2020 May 24;20:186. doi: 10.1186/s12935-020-01273-0 (PMC7247123; doi:10.1186/s12935-020-01273-0)
Supplement: Supplementary file 1 — Additional file 1: Table S1. List of inconsistent cases between pathologists Huang Dan and Qiongyan Zhang for which a third evaluation by pathologist Lei Wang was essential. [file 12935_2020_1273_MOESM1_ESM.docx]

Additional Table S1: List of inconsistent cases between pathologists Huang Dan and Qiongyan Zhang for which a third evaluation by pathologist Lei Wang was essential

| Pathologist | | | | |
| --- | --- | --- | --- | --- |
| Inconsistent cores | Case number | Dan Huang | Qiongyan Zhang | Lei Wang |
| 1 | 4 | - | + | - |
| 2 | 5 | + | - | + |
| 3 | 9 | + | - | + |
| 4 | 12 | - | + | + |
| 5 | 14 | + | - | - |
| 6 | 28 | - | + | + |
| 7 | 36 | + | - | + |
| 8 | 39 | + | - | - |
| 9 | 40 | - | + | - |
| 10 | 45 | - | + | + |
| 11 | 53 | + | - | + |
| 12 | 59 | - | + | + |
| 13 | 62 | + | - | - |
| 14 | 67 | - | + | + |
| 15 | 69 | + | - | + |
| 16 | 71 | - | + | + |
| 17 | 72 | + | - | + |
| 18 | 78 | - | + | - |
| 19 | 84 | + | - | + |
| 20 | 87 | - | + | + |
| 21 | 90 | - | + | + |
| 22 | 94 | + | - | + |
| 23 | 98 | + | - | + |
| 24 | 106 | - | + | + |
| 25 | 111 | - | + | + |
| 26 | 129 | + | - | - |
| 27 | 131 | + | - | + |
| 28 | 149 | - | + | + |
| 29 | 152 | + | - | + |
